# Relationship Between Accelerometer-Based Physical Activity, Sedentary Behavior, and Mental Health in Young Finnish Men

**DOI:** 10.3389/fpubh.2022.820852

**Published:** 2022-02-18

**Authors:** Kaija Appelqvist-Schmidlechner, Jani Raitanen, Tommi Vasankari, Heikki Kyröläinen, Arja Häkkinen, Tuomas Honkanen, Jani P. Vaara

**Affiliations:** ^1^Finnish Institute for Health and Welfare, Mental Health Unit, Helsinki, Finland; ^2^Centre for Military Medicine, Helsinki, Finland; ^3^Urho Kaleva Kekkonen Institute for Health Promotion Research, Tampere, Finland; ^4^Faculty of Social Sciences (Health Sciences), Tampere University, Tampere, Finland; ^5^Faculty of Medicine and Health Technology, Tampere University, Tampere, Finland; ^6^Faculty of Sport and Health Sciences, University of Jyväskylä, Jyväskylä, Finland; ^7^Department of Leadership and Military Pedagogy, National Defence University, Helsinki, Finland; ^8^Department of Physical Medicine and Rehabilitation, Central Hospital of Central Finland, Jyväskylä, Finland

**Keywords:** physical activity, mental health, sedentary behavior, mental well-being, men's health, mental health problems

## Abstract

Healthy lifestyle behaviors including physical activity (PA) have been recognized to contribute positively to mental health. Most of the evidence on relationship between PA and mental health relies on self-reported PA results. Device-based measures on PA or sedentary behavior (SB) are less frequently used in mental health research. The present study aimed at examining the relationship between mental health and PA/SB measured by accelerometers in young Finnish men. The sample consisted of 409 men (mean age 28 ± 7 years), who participated in the military refresher training in Finland. Self-rated mental health was measured with Mental Health Inventory (MHI-5) and short Warwick-Edinburgh Mental Well-being Scale (SWEMWBS) measuring mental health both from the perspective of mental health problems and mental well-being. PA was measured with accelerometer from the perspective of light, moderate, vigorous, and total activity, as well as SB. Linear regression models and compositional analysis were applied. Age, education, marital status, employment status, BMI, alcohol use and smoking were used as covariates. Evidence on relationship between total PA (standardized regression coefficient 0.340; 95% CI 0.022–0.657, *p* = 0.036) and SB (standardized regression coefficient −0.340; 95% CI −0.657 to −0.022, *p* = 0.036) with symptoms of mental health problems was found after adjusting for age, education, marital and employment status. The relationship was marginally significant (*p* = 0.056) after adjusting also for BMI, alcohol use and smoking. No evidence on relationship between PA or SB and mental well-being was found, neither in standard linear regression analysis nor in compositional approach. In our sample of young adult men, PA seemed to have a stronger relationship with symptoms of mental health problems rather than with mental well-being. The findings lead to a conclusion that all PA *per se* may not be independently associated with mental well-being in young adult males and raise the question whether the domain of PA and its context play a critical role in these relationships.

## Introduction

In recent years, there has been increasing understanding for mental health as a valued source of human capital in society and the importance to implement strategies for promoting mental well-being and preventing mental health problems in different populations (1). Mental health is defined as a state of well-being in which an individual realizes his or her own abilities, can cope with the normal stresses of life, can work productively and is able to make contribution to his or her community (2). Mental health is more than just absence of mental health problems, like a diagnosed mental disorder such as depression or anxiety or mental distress without a diagnosed mental disorder. The dual continuum model of mental health views mental health problems and mental well-being as two separate continua rather than as opposite ends of the same continuum. They are related, but distinct dimensions, one indicating the presence or absence of mental health and the other the presence of absence of mental disorder (3). From the public health perspective, it is crucial to identify factors that strengthen mental health and reduce the risk of mental health problems. Research on determinants, such as healthy lifestyle behaviors, including physical activity (PA), that are associated with mental well-being on the one hand and with mental health problems on the other hand, is therefore highly relevant.

Benefits of PA for mental health are well-documented, both from the perspective promoting mental well-being and preventing or dealing with mental health problems. Previous research suggest that PA is positively associated with better mental health (4, 5), decreased likelihood for mental health problems (6–8), protect against the emergence of depression (6, 9–12) and anxiety (13, 14), and that PA can be used in the treatment of depression (15).

Besides PA, there is a growing interest to understand the role of sedentary behavior (SB)—defined as any waking behaviors characterized by energy expenditure ≤1.5 metabolic equivalents while in a sitting or reclining posture (16)—in mental health of populations. Previous studies suggest that the positive association between PA and mental well-being could be hampered by daily sedentary time (17–19). Despite of partly inconsistent results on the relationship between SB and mental health (20, 21), the findings of previous studies tend to indicate that higher level of SB is associated with a higher level of mental health problems both in adults (22–24) and adolescents (25, 26) and decreased mental well-being in adult populations. Similar findings have been found both in studies using self-reported (4, 18) and device-based SB data (27).

Device-based measures on PA and SB are less frequently used in mental health research (17, 20). Most of the evidence on the association between PA/SB and mental health relies on self-reported measurements of PA (9). However, self-reported data on PA may be subject to social desirability, recall, attention, or mood biases (28). Review by Prince et al. (28) found that self-reported levels of PA were both higher and lower than device-based measured levels of PA, which poses a problem for interpreting and comparing results of studies using different measurement methods. Further, self-report methods have been seen as challenging in investigating light intensity activities due to their unstructured nature and dispersion throughout the day (29, 30). Device-based measures have the possibility to provide more reliable estimates of total PA, including SB, and objective measured estimates for light and moderate to vigorous activity. Despite of the methodological differences, evidence on the crucial role of PA for mental health of individuals—both from the perspective of mental health problems and mental well-being—is provided also by studies that have used device-based measures of PA in both adult populations (10, 17, 27) as well as among children (31) and adolescents (32, 33).

Besides limitations in investigating PA, previous research lacks measurement consistency as mental health has been defined and assessed in a variety of ways. Most studies have focused on mental health problems, mostly focusing on one specific disorder such as depression (9, 10, 23) or anxiety (13, 14). Only few studies (34, 35) have used multi-dimensional measures of mental well-being or symptoms of mental health problems and addressed both spectrums of mental health: mental well-being and mental health problems, Bell et al. (34) focusing on the relationship between PA, mental well-being and mental health problems among adolescents using accelerometer-based PA data and Nakagawa et al. (35) focusing on the relationship between different PA intensity and mental health among young adults using self-reported PA data.

In view of existing limitations of studies using self-reported data on PA, the present study aims to investigate the relationship between accelerometer-based PA and SB with mental health. In terms of PA, we investigated this relationship from the perspective of light, moderate, vigorous and total activity, as well as time spent engaging in SB. In terms of mental health, the focus was both on symptoms of mental health problems and mental well-being providing perspective for the whole spectrum of mental health. We hypothesized that low volume of PA and high volume of SB are associated with higher levels of symptoms and lower levels of mental well-being.

## Materials and Methods

The present study is a part of the Finnish Reservist study, and the participants were young adult men who were called up to the military refresher training organized by the Finnish Defense Forces during May-November in 2015. The data of the present study were collected using a self-administered questionnaire about mental health in the beginning of seven refresher training courses, which were carried out in different counties around Finland, and using accelerometers to monitor PA during one week after the refresher training courses. The study participants were informed about the study in the refresher training call-up letter. Participation was voluntary and, of 823 course participants, 776 participated in the study. Of these men, 519 were randomly selected to the follow-up measures of accelerometer PA and 415 met the inclusion criteria of a minimum of 4 days with at least 10 h accelerometer wear time per day. The sample of this study comprises of 409 men (mean age 28 ± 7 years) with data both on PA and mental health. A written consent form was received from all study participants. The study was approved by the ethical committees of the Central Finland Health Care District, and the Headquarters of the Finnish Defense Forces (AM5527).

### Measurements

*Symptoms of mental health problems* were measured with Mental Health Inventory [MHI-5, (36)]. It consists of five items and evaluates symptoms of depression and anxiety in both clinical and non-clinical populations with the following questions: How much of the time during the last month have you been (1) very nervous, (2) felt downhearted and blue, (3) felt calm and peaceful, (4) felt so down that nothing could cheer you up and (5) happy? The response alternatives were all of the time, most of the time, a good bit of time, some of the time, a little of the time or none of the time. The scores were coded and ranged from 0 to 100, higher scores indicating better mental health. The MHI-5 has been seen as sufficiently brief, easy to complete, valid, and reliable for use with different subgroups and in different cultures (37).

*Mental well-being* was measured with the short Warwick-Edinburgh Mental Well-being Scale [SWEMWBS, (38)], a validated (39) instrument monitoring mental well-being in different populations. Respondents were asked to rate their feelings over the previous 2 weeks from 1 (none of the time) to 5 (all of the time) on the following questions: “I have been feeling optimistic about the future,” “I have been feeling useful,” “I have been feeling relaxed,” “I have been dealing with problems well,” “I have been thinking clearly,” “I have been feeling close to other people,” and “I have been able to make up my own mind about things.” Weighted sum score was calculated, higher score indicating better mental well-being.

*Physical activity (PA) and sedentary behavior (SB)* were measured with accelerometer. A waist-worn triaxial accelerometer (Hookie AM 20, Traxmeet Ltd, Espoo, Finland) was used to measure PA and SB. The accelerometer was attached to the right side of the hip with a belt. Subjects were given instructions to wear the accelerometer for seven consecutive days during wake time. The wake time was defined in this study as time spent awake after waking up and getting out of bed until the time going to bed for sleep. The time spent in bed awake is not included in the wake time of the present study (40). The acceleration data were collected at 100 Hz sampling rate and the raw accelerometer data were stored on a hard disk for further analysis. The mean amplitude deviation (MAD) values of the resultant acceleration of the three orthogonal acceleration components were determined in 6 s epochs. The MAD values have been found to be a valid indicator of incident energy expenditure during locomotion (41). The MAD values were converted to metabolic equivalents (METs) for each epoch (42). PA was divided into three intensity categories regarding METs: light physical activity (LPA) 1.5–2.9 MET; moderate physical activity (MPA) 3.0–6.0 MET and vigorous physical activity (VPA) >6.0 MET. Total PA was calculated as the combined amount of light, moderate and vigorous activities. Sedentary time was defined as the time spent in the lying, sitting and standing positions without movement (<1.5 MET) (43). Those who had used the accelerometer at least 4 days were included in the study.

### Statistics

Descriptive statistics and frequency tables were used to explore distribution and frequency of all relevant variables for the study. LPA, MPA, and VPA are reported as percentages of wake time (Table 1). The volume of physical activity/sedentary time was divided into quintiles in order to observe particularly the most and least active group of men. Unadjusted association with mental health variables was analyzed with one-way ANOVA (Table 2). Then, linear regression models were used (Tables 3, 4) and secondly, compositional analysis were applied (CoDaPack-software, v2.03.11) similar to Chastin et al. (35). Compositional analysis takes into account that time is finite during the day and thus time spent in different behaviors are co-dependent. Linear regressions were used to calculate standardized regression coefficients (β) with 95% confidence intervals (CI) p-level set at <0.05 with using unadjusted and adjusted (age, education, marital status, employment status, BMI, use of alcohol, and smoking) models. In compositional analysis, we first re-scaled the original time variables in different behaviors up to 1 (i.e., they varied between 0 and 1 and the sum of them was 1) and secondly, we made an isometric log-ratio (ILR) transformation for these proportions (44). Finally, the new ILR-transformed variables were used as exposure variables in linear regression models, unadjusted and adjusted models (age, education, marital status, employment status, BMI, alcohol use, and smoking) were used (Table 5).

**Table 1:** Characteristics of study participants (*n* = 409).

**Variable**	**Value**
**Education** (***n*** **=** **407), % (*****n*****)**	
Elementary school/vocational school	44 (180)
Upper secondary school/college/university of applied sciences/academic degree	56 (227)
**Marital status (*****n*** **=** **408), % (*****n*****)**	
Widow/divorced/unmarried	47 (191)
Married/cohabitation	53 (217)
**Working status (*****n*** **=** **408), % (*****n*****)**	
Student/unemployed/other	27 (110)
Full-time/part-time employee	73 (298)
SWEMWBS^*^ (*n* = 306), mean (SD)	24.4 (2.9)
MHI-5^**^ (*n* = 409), mean (SD)	78.9 (13.6)
**Sedentary time and physical activity (*****n*** **=** **409), relative to wake time, Mean percentage %, (SD)**	
Total sedentary time (<1.5 MET)	77.4 (8.1)
Total activity (≥1.5 MET)	22.6 (8.1)
Light activity (1.5–2.9 MET)	13.5 (5.2)
Moderate activity (3–6 MET)	8.7 (3.6)
Vigorous activity (>6 MET)	0.49 (0.67)
Moderate-to-vigorous (≥3 MET)	9.2 (3.8)

* *Short Warwick Edinburgh mental well-being scale*.

** *Mental health inventory*.

**Table 2:** Means (SD) of mental health variables by sedentary behavior and physical activity.

	MHI-5^*^			SWEMWBS^**^		
	***N***	**Mean (SD)**	***p***	***n***	**Mean (SD)**	***p***
**Sedentary time (<1.5 MET) (relative to wake time %)**			0.036			0.074
1st quintile	82	81.1 (12.6)		52	24.3 (2.1)	
2nd, 3rd, and 4th quintiles	245	79.2 (13.6)		188	24.5 (2.8)	
5th quintile	82	75.8 (14.0)		66	24.0 (3.8)	
**Physical activity (relative to wake time %)**
Light Activity (1.5–2.9 MET)			0.064			0.186
1st quintile	82	76.0 (14.4)		61	23.8 (3.3)	
2nd, 3rd, and 4th quintiles	245	79.2 (13.2)		194	24.5 (3.0)	
5th quintile	82	80.9 (13.5)		51	24.6 (2.3)	
Moderate Activity (3–6 MET)			0.213			0.419
1st quintile	82	76.6 (14.1)		62	24.4 (4.0)	
2nd, 3rd, and 4th quintiles	245	79.3 (13.5)		187	24.5 (2.7)	
5th quintile	82	80.0 (12.9)		57	23.9 (2.3)	
Vigorous Activity (>6 MET)			0.420			0.525
1st quintile	82	77.1 (15.1)		65	24.1 (2.9)	
2nd, 3rd, and 4th quintiles	245	79.3 (13.0)		179	24.4 (3.0)	
5th quintile	82	79.4 (13.5)		62	24.6 (2.9)	
Moderate-Vigorous Activity (≥3 MET)			0.151			0.471
1st quintile	82	76.3 (14.8)		64	24.1 (3.6)	
2nd, 3rd, and 4th quintiles	245	79.5 (13.0)		187	24.7 (2.8)	
5th quintile	82	79.7 (13.9)		55	23.7 (2.2)	
Total Activity (≥1.5 MET)			0.036			0.074
1st quintile	82	75.8 (14.0)		66	24.0 (3.8)	
2nd, 3rd, and 4th quintiles	245	79.2 (13.6)		188	24.5 (2.8)	
5th quintile	82	81.1 (12.6)		52	24.3 (2.1)	

*p = One-Way ANOVA*.

* *Mental Health Inventory*.

** *Short Warwick Edinburgh Mental Well-Being Scale (n = 306)*.

**Table 3:** Unadjusted and adjusted standardized regression coefficients of sedentary time and physical activity with their 95% confidence intervals (CI) for Short Warwick Edinburgh Mental Well-Being Scale (SWEMWBS).

	**Unadjusted model (*****n*** **=** **306)**		Adjusted model^*^(***n*** **=** **304)**		Adjusted model^**^(***n*** **=** **301)**		Adjusted model^***^(***n*** **=** **301)**	
	**β (95% CI)**	***p***	**β (95% CI)**	***p***	**β (95% CI)**	***p***	**β (95% CI)**	***P***
**Sedentary time (<1.5 MET) (relative to wake time %)**								
Total sedentary time	−0.024 (−0.137 to 0.089)	0.675	0.013 (−0.101 to 0.128)	0.817	0.031 (−0.084 to 0.147)	0.594	0.030 (−0.087 to 0.146)	0.618
1st quintile (ref.)
2nd, 3rd, and 4th quintiles	0.079 (−0.230 to 0.388)	0.615	0.047 (−0.255 to 0.348)	0.761	0.046 (−0.255 to 0.347)	0.763	0.050 (−0.251 to 0.352)	0.742
5th quintile	−0.089 (−0.455 to 0.276)	0.631	0.029 (−0.337 to 0.395)	0.875	0.071 (−0.300 to 0.442)	0.706	0.063 (−0.308 to 0.434)	0.739
**Physical Activity (relative to wake time %)**								
Light Activity (1.5–2.9 MET)	0.067 (−0.046 to 0.179)	0.243	0.018 (−0.100 to 0.135)	0.768	0.001 (−0.117 to 0.118)	0.993	−0.004 (−0.123 to 0.114)	0.944
1st quintile (ref.)
2nd, 3rd, and 4th quintiles	0.238 (−0.051 to 0.526)	0.106	0.079 (−0.207 to 0.364)	0.589	0.068 (−0.216 to 0.352)	0.638	0.086 (−0.198 to 0.371)	0.551
5th quintile	0.279 (−0.093 to 0.652)	0.141	0.114 (−0.267 to 0.494)	0.557	0.095 (−0.284 to 0.474)	0.623	0.098 (−0.283 to 0.478)	0.614
Moderate Activity (3–6 MET)	−0.041 (−0.154 to 0.072)	0.472	−0.046 (−0.155 to 0.064)	0.411	−0.061 (−0.172 to 0.049)	0.274	−0.054 (−0.165 to 0.057)	0.337
1st quintile (ref.)
2nd, 3rd, and 4th quintiles	0.030 (−0.258 to 0.319)	0.835	0.030 (−0.250 to 0.311)	0.832	−0.021 (−0.303 to 0.260)	0.882	−0.002 (−0.285 to 0.280)	0.986
5th quintile	−0.192 (−0.553 to 0.169)	0.295	−0.241 (−0.593 to 0.112)	0.180	−0.298 (−0.652 to 0.057)	0.099	−0.269 (−0.626 to 0.088)	0.139
Vigorous Activity (> 6 MET)	0.019 (−0.110 to 0.148)	0.768	0.009 (−0.115 to 0.132)	0.889	0.018 (−0.108 to 0.144)	0.777	0.033 (−0.095 to 0.160)	0.614
1st quintile (ref.)
2nd, 3rd, and 4th quintiles	0.109 (−0.177 to 0.394)	0.455	0.154 (−0.121 to 0.429)	0.270	0.161 (−0.118 to 0.441)	0.257	0.179 (−0.101 to 0.459)	0.210
5th quintile	0.161 (−0.189 to 0.511)	0.367	0.175 (−0.163 to 0.512)	0.309	0.209 (−0.139 to 0.557)	0.238	0.238 (−0.112 to 0.588)	0.181
Moderate-Vigorous Activity (≥ 3 MET)	−0.039 (−0.152 to 0.074)	0.495	−0.046 (−0.155 to 0.063)	0.409	−0.060 (−0.170 to 0.050)	0.286	−0.051 (−0.162 to 0.060)	0.365
1st quintile (ref.)								
2nd, 3rd, and 4th quintiles	0.198 (−0.085 to 0.482)	0.170	0.188 (−0.088 to 0.463)	0.181	0.145 (−0.132 to 0.423)	0.303	0.146 (−0.131 to 0.424)	0.300
5th quintile	−0.128 (−0.488 to 0.232)	0.484	−0.155 (−0.504 to 0.194)	0.382	−0.202 (−0.555 to 0.150)	0.260	−0.179 (−0.534 to 0.177)	0.323
Total activity (≥ 1.5 MET)	0.024 (−0.089 to 0.137)	0.675	−0.013 (−0.128 to 0.101)	0.817	−0.031 (−0.147 to 0.084)	0.595	−0.030 (−0.146 to 0.087)	0.618
1st quintile (ref.)								
2nd, 3rd, and 4th quintiles	0.168 (−0.114 to 0.450)	0.241	0.017 (−0.260 to 0.294)	0.902	−0.025 (−0.303 to 0.254)	0.861	−0.013 (−0.293 to 0.267)	0.929
5th quintile	0.089 (−0.276 to 0.455)	0.631	−0.029 (−0.395 to 0.337)	0.875	−0.071 (−0.442 to 0.300)	0.706	−0.063 (−0.434 to 0.308)	0.739

* *Adjusted for age, education, marital status, and employment status*.

** *Additionally, adjusted for BMI*.

*** *Additionally, adjusted for smoking and alcohol consumption*.

**Table 4:** Unadjusted and adjusted standardized regression coefficients of sedentary time and physical activity with their 95% confidence intervals (CI) for mental health inventory (MHI-5).

	**Unadjusted model (*****n*** **=** **409)**		Adjusted model^*^(***n*** **=** **407)**		Adjusted model^**^(***n*** **=** **399)**		Adjusted model^***^(***n*** **=** **399)**	
	**β (95% CI)**	***P***	**β (95% CI)**	***p***	**β (95% CI)**	***p***	**β (95% CI)**	***p***
**Sedentary time (<1.5 MET) (relative to wake time %)**								
Total sedentary time	−0.086 (−0.183 to 0.011)	0.083	−0.065 (−0.168 to 0.037)	0.212	−0.054 (−0.160 to 0.051)	0.313	−0.059 (−0.164 to 0.046)	0.269
1st quintile (ref.)								
2nd, 3rd, and 4th quintiles	−0.138 (−0.387 to 0.111)	0.278	−0.173 (−0.426 to 0.080)	0.179	−0.162 (−0.423 to 0.098)	0.221	−0.160 (−0.420 to 0.099)	0.226
5th quintile	−0.392 (−0.698 to −0.087)	0.012	−0.340 (−0.657 to −0.022)	0.036	−0.316 (−0.645 to 0.013)	0.060	−0.320 (−0.648 to 0.008)	0.056
**Physical activity (relative to wake time %)**								
Light Activity (1.5–2.9 MET)	0.091 (−0.006 to 0.188)	0.065	0.068 (−0.036 to 0.173)	0.200	0.058 (−0.049 to 0.165)	0.290	0.055 (−0.052 to 0.162)	0.314
1st quintile (ref.)								
2nd, 3rd, and 4th quintiles	0.230 (−0.019 to 0.480)	0.070	0.117 (−0.140 to 0.375)	0.370	0.113 (−0.148 to 0.374)	0.395	0.123 (−0.137 to 0.383)	0.351
5th quintile	0.356 (0.051 to 0.662)	0.022	0.269 (−0.056 to 0.594)	0.105	0.250 (−0.082 to 0.582)	0.139	0.247 (−0.084 to 0.578)	0.143
Moderate activity (3–6 MET)	0.066 (−0.031 to 0.163)	0.183	0.054 (−0.045 to 0.153)	0.286	0.044 (−0.057 to 0.145)	0.392	0.055 (−0.046 to 0.156)	0.282
1st quintile (ref.)								
2nd, 3rd, and 4th quintiles	0.200 (−0.050 to 0.451)	0.116	0.167 (−0.086 to 0.419)	0.195	0.153 (−0.105 to 0.411)	0.243	0.167 (−0.091 to 0.424)	0.205
5th quintile	0.248 (−0.058 to 0.555)	0.112	0.197 (−0.118 to 0.511)	0.219	0.171 (−0.151 to 0.493)	0.296	0.214 (−0.108 to 0.535)	0.192
Vigorous activity (> 6 MET)	0.022 (−0.089 to 0.132)	0.702	0.019 (−0.092 to 0.130)	0.737	0.017 (−0.097 to 0.131)	0.767	0.033 (−0.082 to 0.147)	0.576
1st quintile (ref.)								
2nd, 3rd, and 4th quintiles	0.162 (−0.089 to 0.413)	0.205	0.192 (−0.058 to 0.441)	0.132	0.188 (−0.068 to 0.444)	0.150	0.207 (−0.048 to 0.461)	0.112
5th quintile	0.166 (−0.142 to 0.473)	0.290	0.184 (−0.124 to 0.491)	0.240	0.182 (−0.134 to 0.499)	0.259	0.228 (−0.089 to 0.544)	0.159
Moderate-vigorous activity (≥ 3 MET)	0.058 (−0.039 to 0.155)	0.240	0.045 (−0.053 to 0.144)	0.363	0.036 (−0.065 to 0.137)	0.484	0.050 (−0.051 to 0.150)	0.333
1st quintile (ref.)								
2nd, 3rd, and 4th quintiles	0.235 (−0.015 to 0.486)	0.065	0.191 (−0.061 to 0.444)	0.136	0.177 (−0.081 to 0.435)	0.177	0.182 (−0.074 to 0.438)	0.164
5th quintile	0.252 (−0.054 to 0.558)	0.107	0.213 (−0.097 to 0.523)	0.178	0.183 (−0.136 to 0.502)	0.260	0.222 (−0.096 to 0.541)	0.171
Total activity (≥ 1.5 MET)	0.086 (−0.011 to 0.183)	0.083	0.065 (−0.037 to 0.168)	0.212	0.054 (−0.051 to 0.160)	0.313	0.059 (−0.046 to 0.164)	0.269
1st quintile (ref.)
2nd, 3rd, and 4th quintiles	0.254 (0.005 to 0.504)	0.046	0.167 (−0.086 to 0.419)	0.195	0.154 (−0.104 to 0.412)	0.242	0.160 (−0.099 to 0.418)	0.225
5th quintile	0.392 (0.087 to 0.698)	0.012	0.340 (0.022 to 0.657)	0.036	0.316 (−0.013 to 0.645)	0.060	0.320 (−0.008 to 0.648)	0.056

* *Adjusted for age, education, marital status, and employment status*.

** *Additionally, adjusted for BMI*.

*** *Additionally, adjusted for smoking and alcohol consumption*.

**Table 5:** Linear regression models estimates (γ) with *p*-values of isometric log-ratio transformed sedentary time and physical activity for Mental Health Inventory (MHI-5) and Short Warwick Edinburgh Mental Well-Being Scale (SWEMWBS).

	**Unadjusted model**		Adjusted model^*^		Adjusted model^**^		Adjusted model^***^	
	**γ**	***p***	**γ**	***p***	**γ**	***p***	**γ**	***P***
**Outcome** **=** **MHI-5**								
*N*	409		407		399		399	
Total sedentary time vs. remaining behaviors	−0.246	0.046	−0.181	0.169	−0.150	0.271	−0.156	0.249
Light activity vs. remaining behaviors	0.203	0.286	0.099	0.621	0.081	0.691	0.038	0.851
Moderate-vigorous activity vs. remaining behaviors	0.043	0.813	0.081	0.656	0.069	0.711	0.118	0.528
**Outcome** **=** **SWEMWBS**								
*N*	306		304		301		301	
Total sedentary time vs. remaining behaviors	−0.134	0.379	−0.041	0.793	0.035	0.828	0.031	0.848
Light activity vs. remaining behaviors	0.407	0.069	0.151	0.514	0.097	0.673	0.064	0.785
Moderate-vigorous activity vs. remaining behaviors	−0.273	0.209	−0.110	0.601	−0.132	0.532	−0.095	0.658

* *Adjusted for age, education, marital status, and employment status*.

** *Additionally, adjusted for BMI*.

*** *Additionally, adjusted for smoking and alcohol consumption*.

## Results

The descriptive analyses revealed that 56% of the participants had at least an upper secondary school education, 53% were married and 73% working full or part time. The mean score of SWEMWBS was 24.4 (SD 2.9) and MHI-5 78.9 (SD 13.6). The participants spend on average 77% of their wake time with sedentary behavior and 23% with PA with different intensity: 14% with light and 9% with moderate-to-vigorous PA (Table 1).

Men in the quintile with most sedentary time had lowest and the quintile with the least sedentary time the highest scores in MHI-5 scale measuring symptoms of mental health problems, which indicates that more sedentary time is related to more symptoms (*p* = 0.036). Accordingly, in terms of total PA in the most active quintile of men, the scores were the highest and in the most passive quintile the lowest indicating that more PA is related to less symptoms (*p* = 0.036). No evidence on relationship between symptoms of mental health problems and light/moderate/vigorous PA was found. Likewise, in terms of mental well-being, neither SB nor PA were related to SWEMWBS scores (Table 2).

According to the findings of linear regression models (Tables 3, 4), a weak relationship was found between SB and total PA with symptoms of mental health problems, but not with mental well-being. The quintile with the most sedentary time had statistical significantly more symptoms compared with the quintile of least sedentary time, also after adjusting for age, education, marital status and employment status (β −0.340, 95% CI −0.657 to −0.022). The quintile with least total PA had more symptoms compared with the quintiles with more PA when adjusting for age, education, marital status and employment status (β 0.340, 95% CI 0.022–0.657). These relationships were marginally significant (*p* = 0.056) after adjusting also for BMI, alcohol use and smoking. In terms of different intensity of PA, relationship was found only in the variable of light PA, but not in the models adjusted for confounding variables. The quintile of men with the least light PA had higher level of symptoms compared with the quintile of men with the lightest PA (β 0.356, 95% CI 0.051–0.662), but only in the unadjusted model.

Regression models on compositional data showed that the unadjusted relationship between the proportion of time spent in SB and symptoms of mental health problems was significant (regression coefficient (γ) −0.25, *p* = 0.046). However, this significant finding disappeared after adjustments. No statistically significant relationship was found between PA or SB with mental well-being (Table 5).

## Discussion

Contrary to expectations, the present study found only a weak relationship between accelerometer-based PA and SB with mental health. A statistically significant relationship was found between SB and total PA with symptoms of mental health problems after adjusting for age, education, marital status, and employment status. These relationships were marginally significant after adjusting also for BMI, alcohol use and smoking. No evidence on relationship between PA or SB with mental well-being was found, neither in standard linear regression analysis nor in compositional approach. Thus, in our sample of young healthy men, physical activity had a stronger relationship with mental health from the perspective of mental health problems rather than from the perspective of mental well-being.

The findings of the present study are not quite consistent with findings of previous research. Various of earlier studies have found a positive association between PA and mental health—both from the perspective of mental health problems (9, 10, 13, 15) and mental well-being (4, 18, 27) and using self-reported (4, 8, 18) and device-based measured (10, 17, 27) PA data. However, this relationship has been seen to be complex and non-linear (17). The differences in the findings of the present study compared with those indicating PA to be associated with better mental well-being and less mental health problems may be explained by methods used in observing PA and mental health. Device-based measures on PA are less frequently used in mental health research (9, 17, 20) and findings from previous studies using device-based data have been contradictory. The meta-analysis by Gianfredi et al. (10) showed a potential protective effect of PA on prevalent and incident of depression, similar to the findings of the study by Choi et al. (11). Further, Bernard et al. (17) found in their cross-sectional study in a representative national sample of adults a positive association between accelerometer-based PA and mental health, daily SB hampering this association. However, mental health was in this study investigated with one single question focusing on self-perceived mental health status without distinction between mental well-being and mental health problems. Evidence on the role of accelerometer-based PA in mental health outcomes exist also in studies using samples of children and adolescents (25). However, Konopka et al. (20) found no association between accelerometer-based SB or PA with depression symptoms in men. Similarly, a cohort study by Bell et al. (34) found no evidence of an association between accelerometer-based PA, mental well-being and symptoms of mental disorders, but the sample consisted of adolescents and the findings are therefore not comparable with the findings of the present study. Zhang et al. (45) examined the bidirectional associations between moderate to vigorous PA and depressive symptoms and investigated also the differences in the observed associations using self-reported and device-based measured PA data. They found an inverse bidirectional association between self-reported PA and depressive symptoms. Higher levels of vigorous intensity PA at baseline, but not moderate intensity PA were associated with lower levels of depressive symptoms at 10-year follow-up. However, no association was found between accelerometer-based PA estimates and depressive symptoms. The relationship between PA, SB and mental health seems to be very complex with various mediating factors, such as health behavior including BMI, smoking or use of alcohol, like presented in the current study.

In terms of SB, the findings were likewise unexpected compared with the findings of previous studies. The present study found no evidence on relationship between SB and mental well-being and only a weak relationship between SB and symptoms of mental health problems, although SB has been suggested to be associated with a higher risk of mental health problems in adult populations. However, these studies have most commonly used self-reported PA data (4, 20–22). Also contradictory findings have been reported, for example, by the review of Teychenne et al. (21) and by the study of Konopka et al. (20) who examined the association between accelerometer-derived SB and depressive symptoms over 4 years follow-up and found no association between SB and depressive symptoms. In contrary to the present study, previous studies have commonly focused on specific mental health problems and the perspective of mental well-being has been less frequently in the focus of investigations.

The present study found no evidence on relationship between different intensity levels of PA and mental health—neither from the perspective of mental health problems nor from the perspective of mental well-being, in contrary to the findings of previous studies. It is difficult to explain this result, but it might have something to do with the use of different methods in measuring PA and the dominant role of self-reported PA data in previous studies. Asztalos et al. (46) suggest that the relationship between self-reported PA and mental health varies across activity intensity levels and dimensions of mental health among women and men. Previous studies using self-reported PA data have reported the crucial role of moderate-to-vigorous PA in the promotion of mental well-being in men (35, 46, 47). More research using device-based PA data is needed.

The findings of the present study may be explained by previous research indicating that the domain of physical activity plays an important role in the relationship between PA and mental health. Relying on findings from our previous study with the same sample of men using self-reported PA data (48), the findings of the present study highlight potentially the crucial role of domain and context of PA for the mental health of individuals. The importance of especially leisure-time PA in the relationship between PA and mental health is well-documented (48–51) and may be explained, for example, by voluntary nature, own preferences, enjoyment and possibilities for social interactions (7, 52). A number of other previous studies using self-reported PA data (53–55) have shown that activities that are more likely to be practiced in group, are strongly associated with lower depressive symptoms and better mental well-being, especially in men (55).

The mechanism underlying the association between PA, SB, mental well-being and mental health problems is known to be complex (56, 57). Further, the findings of different studies depend also both on methods used in measuring PA as well as on the choice whether mental health is assessed from the perspective of mental well-being or mental health problems. There is yet limited evidence on mechanisms between physical activity and mental health explaining the findings of the present study compared with observations of previous research. Integrative and more complex models are needed combining different hypothesis on potential underlying mechanisms (for example neurobiological, psychosocial and behavioral) in order to widen our understanding on the relationship between PA and mental health.

### Strengths and Limitations of the Study

Strengths of this study include the use of accelerometer-based measurements on PA and validated scales on mental health covering both mental well-being and symptoms of mental health problems. Use of accelerometer allows the objective measurement of PA and SB, while most other studies have used self-reported data which might lead to information bias. We were also able to control for a wide range of possible confounding factors which can be seen as a strength of the study.

The study also had several limitations. First, the cross-sectional study design precludes inferences about causal relationships. Second, some limitations should be noted in terms of using accelerometers. Selection bias and missing data in terms of wearing accelerometers during the time period required could have influenced the results but it is difficult to estimate in which way. Further, accelerometers are known to have difficulties in recording accurately certain activities, such as cycling or weightlifting (58). It is also possible, that wearing the accelerometer have influenced normal PA behavior during the observed time period. In terms of SB, accelerometers are unable to detect contextual information about different types of activity and sedentary time. For example, SB can be mentally active (like working or reading) or passive (like watching television) and findings from previous studies suggest that mentally active SB is associated with less mental health problems (59). Third, the sample consisted of young healthy men, which has to be taken in consideration before generalizing the findings for entire male population. Previous studies have shown that men exempted from the military service (and consequently excluded also from the reservist training courses) have more psychosocial problems compared to those men who complete the service (60). Thus, men with poorer psychosocial and health status are underrepresented in the sample.

## Conclusions

We found evidence only for a weak relationship between accelerometer-based PA and mental health in our sample of young men. Lower total PA and higher level of SB were associated with more symptoms of mental health problems. No evidence on relationship between PA or SB with mental well-being was found. The present results highlight that among young healthy men, SB and total PA may be weakly associated with mental health, especially from the perspective of mental health problems, whereas different dimensions of intensity were not associated. Nevertheless, all PA *per se* may not be independently associated with mental health in young adult males and raise the question whether the domain of PA and its context play a critical role in these relationships, especially from the perspective of mental well-being.

## Implications for Future Research

PA might influence mental health through a variety of psychosocial and neurobiological mechanism and respectively, mental health may have an impact on physical activity of individuals. More research is needed to understand the mechanisms of this relationship. It would be an interesting topic for future research to differentiate between accelerometer-based leisure-time and occupational PA and between mentally active and passive sedentary time, paying in this way more attention to the context of PA and SB. Research is needed both from the perspective of mental health problems and mental well-being.

## Data Availability Statement

The raw data supporting the conclusions of this article will be made available by the authors, without undue reservation.

## Ethics Statement

The studies involving human participants were reviewed and approved by Ethical Committees of the Central Finland Health Care District, and the Headquarters of the Finnish Defence Forces (AM5527). The patients/participants provided their written informed consent to participate in this study.

## Author Contributions

The study is part of the Finnish Reservist 2015 Study led by HK. JV collected the data with the assistance of research assistants. JR conducted the statistical analysis and KA-S led the writing process. KA-S had final responsibility for the decision to submit for publication. All authors contributed to the study design and methodology, provided critical revisions to the manuscript, accepts responsibility for the contents of the article, read, and approved the final version submitted.

## Conflict of Interest

The authors declare that the research was conducted in the absence of any commercial or financial relationships that could be construed as a potential conflict of interest.

## Publisher's Note

All claims expressed in this article are solely those of the authors and do not necessarily represent those of their affiliated organizations, or those of the publisher, the editors and the reviewers. Any product that may be evaluated in this article, or claim that may be made by its manufacturer, is not guaranteed or endorsed by the publisher.
